# Fuel consumption rate and emissions variability in waste collection services routes: case study of Cascais Ambiente

**DOI:** 10.1007/s11356-023-29045-z

**Published:** 2023-08-02

**Authors:** Vitor Sousa, André Drumond, Inês Meireles

**Affiliations:** 1 CERIS, Department of Civil Engineering, Architecture and Environment, Instituto Superior Técnico, Universidade de Lisboa, Av. Rovisco Pais, 1049-001 Lisbon, Portugal; 2 EMAC, Cascais Ambiente, Cascais, Portugal; 3 RISCO, Department of Civil Engineering, University of Aveiro, Campus de Santiago, 3810-193 Aveiro, Portugal

**Keywords:** Waste collection, Mixed waste, Waste generation pattern, Service performance, Fuel consumption

## Abstract

In the design waste collection systems, it is common practice to use a constant specific fuel consumption (e.g. litres per amount of waste collected or distance travelled). This is also the approach used in many cases for fleet management, namely, for decision-making on more fuel-efficient equipment acquisition. However, the specific fuel consumption is not constant and there are spatial and temporal variations. Accounting for this variability becomes relevant if a more refined cost or environmental optimization is intended. The present research effort evaluates the energy intensity of the waste collection service in the Cascais municipality, reporting the differences and the magnitude of the variability for the mixed waste collection service. Statistically significant differences are found between the circuits, the trucks, months of the year and days of the week. It is discussed that extrapolating average fuel consumption rates for decision-making in new waste collection systems may be prone to substantial error.

## Introduction

In addition to the contribution to environmental and public health preservation, solid waste collection is a key piece in the chain towards circular economy in any modern community. The capability to adequately streamline the waste fluxes from their source of origin is a cornerstone to enable the transformation of residues into raw material. Large waste producers are easier to tackle from a logistics point of view, since the spatial spread of the waste generation point is well defined and the waste composition and amounts are more uniform over time. Municipal solid waste, on the other hand, represents a logistic challenge. The spread of the waste generation points, along with the spatial and temporal variation of the composition and amounts of waste generated, builds up to create a problem for which there are multiple possible solutions and the absolute optimal may be difficult to identify and/or may be subjective to the value function defined.

The complexity underlying municipal waste collection is also reflected in terms of the waste management cost proportion. Solid waste collection has been reported to represent between 50 and 70% of the total waste management costs (Sonesson 2000; Dogan and Duleyman 2003; Ghose et al. 2006; Tavares et al. 2009; Sousa et al. 2018). This cost split will depend on the characteristics of the waste producers and the solutions implemented for both the collection and treatment/disposal of the waste. However, the waste treatment/disposal costs may not be just the investment and operational expenses since they are, in many cases, directly affected by policies (e.g. taxes over landfill). Waste collection, on the other hand, tends to reflect the expenses required for providing the service.

Regarding waste collection, fuel costs represent one of the most important categories of costs (e.g. Sousa et al. 2018). Furthermore, fuel costs are intrinsically variable, whereas other cost categories (e.g. labour) are fixed and therefore constant for a given was collection solution. This has led several authors to research this topic of fuel consumption in waste collection from characterisation and/or optimization perspectives.

Within the former group, there have been studies comparing fuel consumption between rear and side-loader waste collection vehicles (e.g. Agar et al. 2007; Ivanič 2007; Thiruvengadam et al. 2010; Sandhu et al. 2015) and between circulation and compactor operation/idling (Bender et al. 2014). In Portugal, an average fuel consumption of 3.96 L/t was estimated for mixed waste collection in the city of Porto (Teixeira et al. 2014b). In additional studies specific to waste collection vehicles, there are also a number of other research efforts on fuel consumption rates for heavy-duty vehicles in general (e.g. USEPA 2002a,b; Ahn and Rakha, 2008; Clark et al. 2002; DEFRA 2005; Eisted et al. 2009; Fruergaard et al. 2009; NRC 2009; Faris et al. 2011; Demir et al. 2014; EPA 2016). Recently, Golbasi and Kina (2022) developed a simulation model accounting for road characteristics, load and precipitation. Indirectly, it is also possible to estimate fuel consumption from models developed for emission estimation (e.g. Liu 2015, USEPA 2016). However, it is important to recall that waste collection is a quite unique use of heavy-duty vehicles that may compromise the use of such models. For instance, in the USA, the fuel consumption was found to be higher for waste collection than for the average heavy-duty vehicles use (76.9 l/100 km versus 33.3 l/100 km) (Gordon et al. 2003; Huai et al. 2006; Nguyen and Wilson 2010).

Optimization-related studies have used deterministic and stochastic approaches. Deterministic approaches adopt average waste generation patterns and seek to optimise the collection schedules and routes using GIS-based models (e.g. Ghose et al. 2006; Tavares et al. 2009; Benitez-Bravo et al. 2021), time/space correlations (e.g. Sonesson 2000; Everett and Riley 1997), scenario analysis (Höke and Yalcinkaya 2021), multicriteria tools (e.g. Amal et al. 2020) or similar methodologies. Stochastic approaches attempt to account for the time and spatial variability of waste generation, addressing dynamic collection scheduling and routing as an alternative to static combined with the use of level sensors within the containers (e.g. Johansson 2006).

Most studies in both of the previous groups resort to data collection and statistical analysis to estimate fuel consumption of waste collection vehicles. Within the scope of waste collection optimization, some authors (e.g. Mohsenizadeh et al. 2020; Franco et al. 2022) have also estimated fuel consumption by simulating the vehicle operation using more complex models, such as the Comprehensive Modal Emission Model developed by Barth et al. (2005).

Fuel consumption and emissions are closely related, existing physical-based models relating both. For instance, the carbon balance model is an example of an approach to estimate fuel consumption from gas emissions. Fuel consumption (and gas emissions) depend on factors that are (Zhou et al. 2016): (i) travel related, (ii) weather related, (iii) vehicle related, (iv) roadway related, (v) traffic related and (vi) driver related. Considering the growing importance given to gas emissions (e.g. climate changes, air quality), the focus has been more on the gas emissions than on the fuel consumption. Based on the classifications proposed by Boulter et al. (2007) and Barati and Shen (2016), emission modelling approaches can be grouped into three main categories of increased level of detail: (i) aggregated, (ii) modal and (iii) simulation. The aggregated approach is the simplest one and emissions are modelled based on the general specifications of the vehicle (Boulter et al. 2007). This approach only provides rough estimates, but serve as basis for the more complex models and still exist nowadays as independent tools or incorporated into more complex models. Some of the aggregated models developed are NONROAD, Nonroad Engines, Equipment, and Vehicles (incorporated into MOVES, MOtor Vehicle Emission Simulator); NMIM, National Mobile Inventory Model (incorporated into MOVES); MOBILE; EMFAC; OFFROAD; CALINE, California Line Source Model, for the USA; and the NAEI, National Atmospheric Emissions Inventory, for Europe. Modal models embed the characterisation of the main operational modes of the vehicles, providing detailed results that can take into account the effects of engine size, engine power, average speed, traffic conditions and other aspects. Authors such as Grote et al. (2016, 2018) or Smit et al. (2010) distinguish between average speed (e.g. COPERT, Computer Programme to calculate Emissions from Road Transport; DMRB, Design Manual for Roads and Bridges, for Europe; COPERTAustralia, for Australia), traffic situation (e.g. HBEFA, Handbook of Emission Factors for Road Transport, from Europe) and traffic variable (e.g. TEE-KCF, Transport Energy and Environment–Kinematic Correction Factor, for Europe). By considering the driving pattern at refined time scales (e.g. second), simulation models have the potential to estimate emissions more accurately. Simulation model approaches can be speed-based (e.g. MODEM; ADVISOR, ADvanced VEhicle SimulatOR, for the USA; DGV, digitised Graz, for Europe) or power-based (e.g. CMEM, Comprehensive Modal Emissions Model, for the USA; VeTESS, VEhicle Transient Emissions Simulation Software; PHEM, Passenger car and Heavy duty Emissions Model, for Europe). Aggregate and modal models require inputs that can be broadly described as traffic variables whereas simulation models require the driving pattern of an individual or a class of vehicles. These comprehensive models reveal that the vehicle fuel consumption is a complex problem and adopting constant values may incur in substantial error.

The various studies related to these topics (waste collection costs, waste collection optimization, fuel consumption and gas emissions) confirm that the optimal configuration and overall performance will depend significantly on the local context. Still, several studies model waste collection fuel and emissions based on constant values (e.g. Yaman et al. 2019). This does not necessarily imply a single constant value, with the models considering variable fuel consumption rates for distinct stages of the waste collection service (e.g. collection, transportation, idling). It means, however, that for each waste collection circuit, the fuel consumption is a constant.

However, the fuel consumption in a given circuit varies each time the service is carried out. This is due to variety of drivers, including traffic and weather conditions, human aspects (e.g. see González et al. (2021) for the influence of the driving style) and the effective waste generated in each period. Within this framework, the present research effort is aimed at disclosing the variability of the specific fuel consumption between and within the waste collection circuits of a single waste collection utility. In the literature review performed, the only study addressing this issue was done by Teixeira et al. (2014a), but the authors only performed a unidimensional analysis (hypothesis testing). This approach provides some insight, but comparing groups individually (e.g. type of containers) implies the assumption that all other factors are equal within each group (e.g. type of trucks, circuit configuration, traffic and weather conditions, topography), which is rarely true. Using the municipality of Cascais as a case study, the variability of the distance, time and waste collected for mixed waste collection circuits is analysed and the specific fuel consumption variation estimated. In addition to the unidimensional statistical analysis, a multidimensional statistical analysis is also performed resorting to multiple linear regression and classification tree. Along with the spatial variability (differences between the waste collection circuits), temporal variability is also found between months of the year and the days of the week. The trucks used, which represent a proxy of the driver, also affect the fuel consumption performance.

In addition to the present section, introducing the topic and presenting the relevant literature review, the document includes three more sections. In the “Case study” section, the case study is detailed in two subsections, starting by presenting the case study and the background studies (“Presentation and background”) and then detailing the data and methods used (“Data and methods”). In the “Results and discussion” section, the results are presented and discussed in three subsections: (i) the “Descriptive analysis” section presents the descriptive statistics, (ii) the “Unidimensional analysis” section reports on unidimensional analysis results and (iii) the “Multidimensional analysis” section presents the statistical models developed in the multidimensional analysis. Finally, the “Conclusions” section presents the conclusions.

## Case study

### Presentation and background

**Fig. 1:**
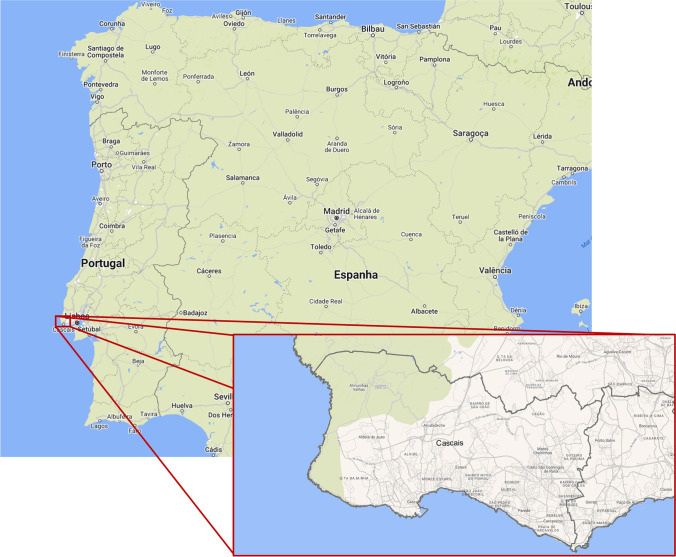
Location of the municipality of Cascais

Located roughly 30 km of Lisbon, Cascais municipality is still within the metropolitan area of the capital of Portugal. Due to the coast shape, the municipality is limited by the Atlantic Ocean on the south and west and by Sintra and Oeiras municipalities on the north and east, respectively (Fig. 1). The population increased from 181,440 inhabitants, in 2003, to 211,714, in 2018, spread over an area of 97.1 km^2^. The population is not uniformly distributed over the territory, with a higher concentration on the southern and eastern regions when compared with the north and west. With a purchasing power per capita over 20% higher than the national average, the municipality of Cascais is one of the richest in Portugal. In 2015, there were only 9 municipalities from a universe of 308 with higher purchase power per capita. The gross domestic product in the Lisbon metropolitan area is roughly 25% higher than the next region on the ranking.

In 2018, the total amount of municipal solid waste collected in Cascais was over 141,000 t. This corresponds to approximately $667\;\mathrm{kg}/\mathrm{hab}\cdot\mathrm{year}$. This amount is higher than the national average of less than $500\;\mathrm{kg}/\mathrm{hab}\cdot\mathrm{year}$, consistent with the relation between wealth and waste generation.

**Fig. 2:**
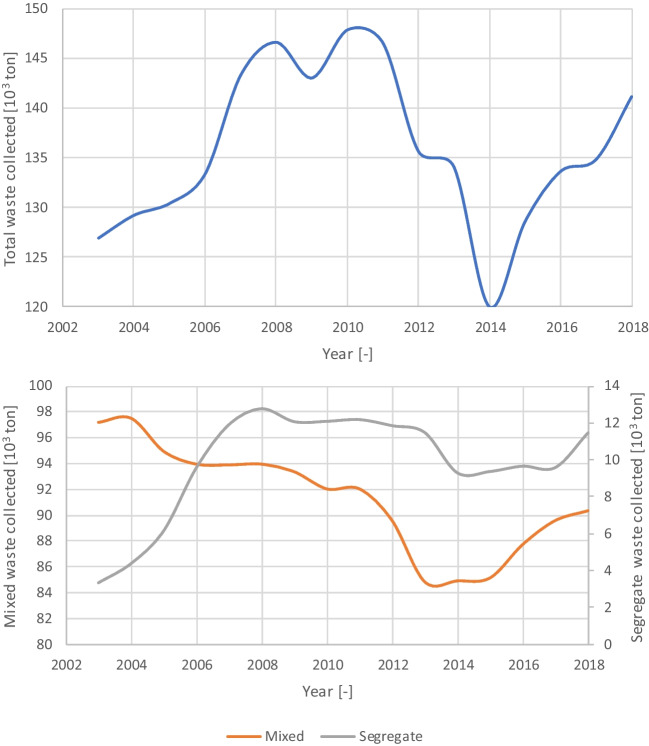
Evolution of the total (top), mixed and segregate (bottom) waste collected in Cascais

This relation is also depicted when analysing the evolution of the total municipal solid waste collected over time (Fig. 2 top). In the economic context, in particular the complete transition to the euro in 2002 and the 2008 world economic crisis, the first marked a period of economic growth, with Portugal benefiting from structural funds from the European Union. The second resulted in the public finance intervention program negotiated with ECB (European Central Bank) and IMF (International Monetary Fund) between 2011 and 2017. Evaluating the evolution of the amounts of mixed and segregate waste collect (Fig. 2 bottom), the pattern is explained also by the economic context, but also by the changes in the service. The creation of a water and waste sector regulator brought new demands for the waste service. In particular, the limits of 200 m and 100 m for the maximum distance between any residence and a segregate and mixed container, respectively, explain the transfer from mixed to segregate waste collection observed between 2003 and 2008 (date when the rule became mandatory).

In both cases, the population increase of nearly 18% between 2003 and 2018 is also relevant for the analysis, especially when evaluating the global impact of the economic context. Analysing the results in per capita waste generation instead of total waste generation reveals a stronger influence from the 2008 economic crisis on the amount of waste generated.

The mixed waste collection service is organized into 19 circuits (ind_100, ind_101, ind_102, ind_111, ind_120, ind_121, ind_130, ind_131, ind_140, ind_141, ind_150, ind_151, ind_160, ind_161, ind_170, ind_171, ind_181, ind_191, ind_200) that operate every day of the week all year. The areas of the circuits are substantially distinct, since the urbanisation density varies within the municipality. The circuits ind_120, ind_121, ind_130, ind_141 and ind_160 cover the west of the municipality, and the circuits ind_161, ind_171 and ind_181 cover the east. The remaining cover the middle, with circuits ind_100 and ind_140 in the north and the rest in the south. This concentration of the circuits in the south in the middle of the municipality matches the population concentration. The circuits were designed based on expert knowledge to allow collecting all the containers within a shift (4 h) and considering the truck limit (in Cascais, roughly 330 to 340 800-l containers are possibly collected by a single truck of 16 m^3^ considering the typical daily fill level). The circuits have been fine-tuned over the last almost 20 years of the operating the service. Also, presently, the automation of the service allows for dynamic operation, in which the containers with the limits between the circuits may be collected in any of the circuits depending on the operation status in each day (e.g. duration of the shift, load level of the truck).

There are waste collection trucks of different brands, but they all have a capacity of 16 m^3^ and 26 t. They are also equipped with a container washing system with 600 l of water. The exceptions are the vehicle numbers 90, 92, 117, 118, 124 and 125. These trucks are from the segregated waste collection service (paper and plastic) and have lower payload (20 t). Their use for mixed waste collection is associated with some unusual circumstance occurring (e.g. vehicle breakdown or unavailability, peak waste generation, for instance, during public events in the municipality).

The detailed cost breakdown structure of the municipal solid waste collection service in the municipality of Cascais, Portugal, was presented in Sousa et al. (2018). Collection was found to represent over 90% of the total cost (the remaining was on street containers), with the operation accounting for 87 to 96% of the collection costs depending on the waste stream. Within the operational costs, labour and maintenance are the largest components, with 45–72% and 12–23%, respectively. Ranging from 9 to 17%, the fuel costs come in the third place and weigh more than the acquisition cost. However, fuel costs are structurally distinct from the other major cost components, since they are intrinsically variable with the collection circuit characteristics (e.g. length of the circuit, number of stops, amount of waste collected, road features) and driving pattern. Acquisition and labour costs are, for a given waste collection service solution, fixed costs. Maintenance costs have an important portion that is fixed (e.g. regular maintenance) and a variable fraction that is indirectly dependent on the circuit characteristics and driving pattern considering that it may affect the durability of the collection vehicles.

### Data and methods

In 2017, EMAC was one of the first organisations in Portugal certified according to the ISO 55000:2014 family of standards and the first on the waste sector. This was accompanied by several improvements within the organisation and the service provided, particularly regarding the amount and quality of the information collected. Pertaining to the present research, the relevant improvements included the development of a comprehensive database recording the duration, the distance and the amount of waste collected per shift and collection circuit, along with all the stops made and the team doing the service. This was done by implementing a mobile automation system comprising the real-time global positioning system (GPS) tracking of the collection vehicles and tagging all containers with radio frequency identification (RFID) tags. This complemented the weighting of the collection vehicles at the waste treatment plant and the fuel purchase records that was already done previously individually for each collection truck in each discharge at the waste treatment plant and in each fuel refill.

This data is available since the beginning of 2018, along with the number and position of the collections made, the stops for unforeseen situations and the containers washed. However, improvements in terms of quality control are being developed and implemented. For instance, the records of the washings are still done manually and are therefore susceptible to human error. Also, the RFID tags and the antennas were found to be prone to failure, either by failing to detect the tag, personnel forgetting to move the tag when a container was replaced or incomplete record due to frequent damage of the antennas. The RFID solution is currently being replaced by the automatic record of the operation of the container loading system in the trucks. This option is more accurate in measuring the number of containers collected, but it does not provide information on which container is collected when there are several in the same location.

All this data is digitally stored in a database developed at EMAC that gathers the data from the different monitoring systems (e.g. GPS and container loading operation automatically recorded in each truck, truck weighting at the waste treatment plant). For this research, the full dataset of 2019 was analysed.

The continuous variables retrieved from the dataset were the number of discharges, the distance covered, the amount of waste collected, the duration of the collection service and the fuel consumed per shift. These variables were then used to estimate additional indicators, namely, (i) the distance and waste collected per discharge and (ii) the fuel consumption and duration amount of waste and per distance. These correspond to the dependent variables to analyse.

As predictors (independent variables), a series of categorical nominal variables were chosen characterising the service conditions (collection circuit and the truck used) and the waste generation pattern (month and the weekday). The mixed waste collection service is organized into 19 circuits and a total of 26 different trucks were used in 2019. Some trucks were only used sporadically when the mixed waste collection trucks were not available. The various circuits are a proxy of differences such as the distance between collection points, topography, traffic conditions, amongst other aspects. The trucks are a proxy of the driving style, since the vehicles dedicated to the mixed waste collection are quite similar in terms of characteristics. The month (12 months of the year) and the weekday (the 7 days of the week) are aimed at capturing the waste generation dynamics, in particular the yearly and weekly seasonality.

**Fig. 3:**
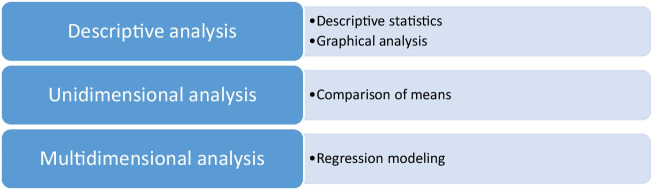
Methodology of analysis

The methodology of analysis used in this research is depicted in Fig. 3. The descriptive analysis is aimed at presenting an overview of the data by presenting some descriptive statistics (e.g. mean, mode, median, standard deviation) and some graphical analysis (boxplots of the continuous dependent variables per group of the independent categorical variables). The unidimensional analysis is aimed at presenting some insight on the data by conducting an analysis of variance (ANOVA) to test the statistical significance of the differences of the continuous dependent variables between the groups of the categorical independent variables. In a first stage, the differences between the amount of waste per circuit are evaluated in overall terms and then per month of the year and each weekday separately. Then, the differences in terms of fuel consumption are assessed. Since in a unidimensional analysis the effect of one predictor may be masked by the effect of another, a multidimensional analysis is performed resorting to regression modelling (ordinary least squares regression and classification trees regression) to assess the influence of each prediction while controlling the remaining.

Before carrying out some of the analysis, in particular the regression and classification tree modelling, the data was screened to remove other incomplete or incorrect data. The screening was done by (i) excluding cases with missing data, in particular in terms of weight collected, distance and fuel consumption, and (ii) excluding cases with distances less than 30 km. The reason underlying the exclusion of circuits with distances under 30 km is explained by the fact that the distance between the EMAC headquarters and the waste treatment plant is roughly 6.5 km. Consequently, the minimum possible distance in a circuit would be around 13 km, corresponding to going from EMAC headquarters to the waste treatment plant and then returning. Additionally, these two facilities are located in less urbanised areas, making it almost impossible to collect a load of waste in the way from EMAC headquarters to the waste treatment plant. After evaluating the circuits, it was found that 30 km would be a good approximation for the minimum distance. Furthermore, not all variables available in the dataset were used. Herein, only the waste collection circuit, truck used, date of the collection service, waste collected, distance covered and fuel consumed were considered. The dataset also includes the number of stops and containers collected

## Results and discussion

### Descriptive analysis

Some basic statistics of the dataset are presented in Table 1. In terms of average values, there are two discharges per shift. This supports even further the option to remove the cases with distances less than 30 km, since two discharges imply a minimum distance of 26 km per shift.

**Table 1:** Statistics of the mixed waste collection data

Statistics		Number of discharges (-)	Distance (km)		Waste collected (kg)		Fuel consumption			Duration		
			Per shift	Per discharge	Per shift	Per discharge	Per shift (l)	Per weight (l/tonne)	Per distance (l/100 km)	Per shift (min)	Per weight (min/tonne)	Per distance (min/km)
Mean		1.91	74.43	40.80	12,772.97	6782.32	74.89	6.28	100.40	344.20	28.41	4.73
Std. error of the mean		0.00	0.13	0.12	30.14	12.62	0.19	0.03	0.17	0.48	0.09	0.01
Median		2.00	74.50	38.60	12,980.00	6740.00	73.60	5.66	96.38	348.00	26.83	4.62
Mode		2.00	78.00	40.20	12,900^a^	7180.00	73.30	5.00	100.00	352.00	25.00	5.00
Std. deviation		0.39	13.02	12.19	3001.47	1257.01	19.19	2.69	17.21	47.98	8.69	0.95
Skewness		1.46	0.58	2.01	0.67	0.16	1.13	3.02	1.38	2.25	6.97	4.68
Std. error of skewness		0.02	0.02	0.02	0.02	0.02	0.02	0.02	0.02	0.02	0.02	0.02
Kurtosis		36.23	15.61	5.09	9.64	0.32	4.84	25.19	1.87	52.62	150.21	53.80
Std. error of kurtosis		0.05	0.05	0.05	0.05	0.05	0.05	0.05	0.05	0.05	0.05	0.05
Minimum		1.00	30.00	4.10	1240.00	1240.00	20.70	0.51	35.92	34.00	0.00	0.00
Maximum		9.00	276.70	138.35	55,120.00	12,820.00	269.30	51.69	147.69	1425.00	311.29	23.17
Percentiles	25	2.00	67.30	33.95	11,040.00	5970.00	64.80	4.63	91.76	328.00	23.72	4.24
	50	2.00	74.50	38.60	12,980.00	6740.00	73.60	5.66	96.38	348.00	26.83	4.62
	75	2.00	81.10	42.50	14,660.00	7570.00	83.30	7.18	104.77	364.00	31.10	5.11

^a^Multiple modes exist. The smallest value is shown

In terms of duration, it was found that it takes almost 30 min to collect a tonne of mixed waste (collection speed of 2.1 t/h) and less than 5 min to travel 1 km (travel speed of 12.7 km/h). The collection speed and travel speed are within the range of values reported by Santos (2011), for Lisbon, and by Teixeira et al. (2014a), for Porto.

The average fuel consumption per amount of waste collected is 50% higher than the value reported by Teixeira et al. (2014b), which is consistent with the higher degree of urbanisation of the municipality of Porto when compared with the municipality of Cascais. Higher population densities also imply more concentrated waste generation, enabling shorter collection circuits to collect the same amount of waste. The 6.28 l/t observed in Cascais is within the range of values reported by Larsen et al. (2009), but substantially higher than the 1.41 l/t, for drop-off, and 1.18 l/t, for street-side containers, reported by Teixeira et al. (2014a) for Porto. This difference is partially explained by the fact that in Porto, the distance covered to collect a tonne of waste is just 2.0–2.2 km, whereas in Cascais is above 6.0 km.

On the other hand, the fuel consumption in terms of distance is 30% higher than reported for the USA. Considering that European communities in general are more concentrated, which can be largely explained by being older and still preserving mediaeval reminiscences within the city spatial organisation, the waste collection circuits are shorter in Europe. Since a large portion of the fuel is consumed during the loading operations and assuming an equivalent number of stops, the fuel consumption per kilometer will tend to be higher in more concentrated communities (the consumption per stop is equivalent but the distance travelled smaller), such as the ones existing in the municipality of Cascais.

**Fig. 4:**
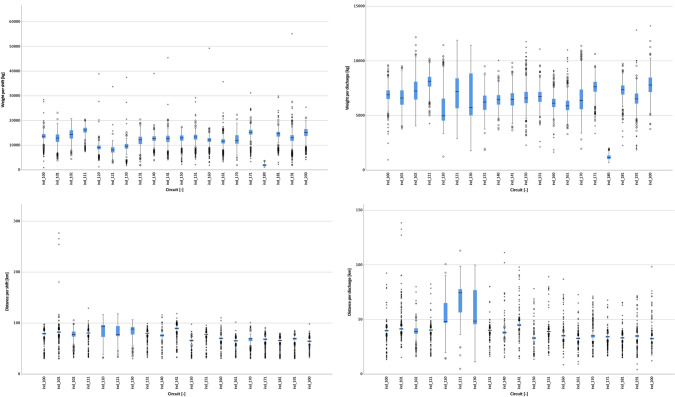
Boxplot of each waste collection circuit in terms of amount of waste collected per shift (top left), waste collected per discharge (top right), distance travelled per shift (bottom left) and distance travelled per discharge (bottom right)

**Fig. 5:**
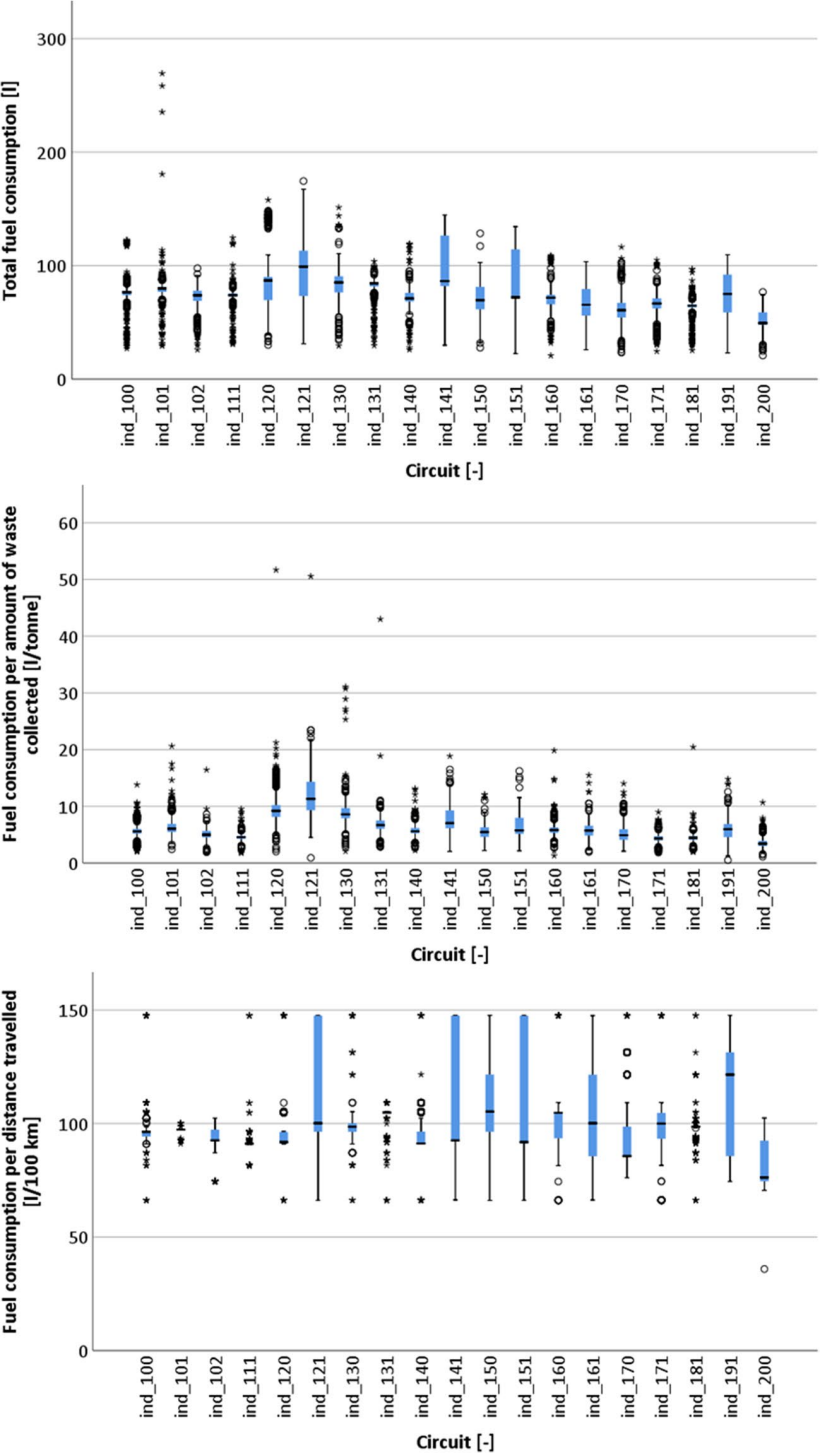
Boxplot of each waste collection circuit in terms of fuel consumption per shift (top), per amount of waste collected (middle) and distance travelled (bottom)

The boxplot presented in Fig. 4 (and also in Fig. 5) indicates the interquartile range (Q1, quartile 1, or percentile 25%, to Q3, quartile 3, or percentile 75%), by a blue box with a horizontal black line inside representing the median. The bars extending above and below indicate the Tukey criterion for outlier identification (values higher than Q3 + 1.5*Q3 − Q1 or lower than Q1 − 1.5*Q3 − Q1). The circles indicate cases classified as outliers, while the stars identify extreme outliers (values higher than Q3 + 3*Q3 − Q1 or lower than Q1 − *Q3 − Q1). A significant variability can be observed both in terms of the amount waste collected and distance travelled per shift and discharge for all collection circuits. The circuits ind_120, ind_121 and ind_131 appear to have a slightly distinct pattern. This is explained by the fact that the circuits in the western areas of the municipality of Cascais are most distant from the waste treatment plant located in the northeast of the municipality. These areas are characterized by a sparse urbanisation comprised mostly single-family houses belonging to the wealthier residents in the municipality. As a result, the distance travelled is high, but the amount of waste collected is not so much. In fact, in many cases, only one discharge is done per shift as a result of the combination of distance (time spent travelling) and the amount of waste generated in the area.

### Unidimensional analysis

The comparison of mean values of distance travelled and amount of waste collect per shift and discharge was done resorting to an ANOVA. The results confirm two aspects: (i) the variance is not homogeneous between circuits and (ii) there are statistically significant differences between almost all circuits. The Levene test indicates that the homogeneity of variance assumption underlying the ANOVA is violated (*p*-value < 0.001 in all cases). So Table 2 presents the results of the Welch and Brown-Forsythe robust tests of equality of means, which confirm the traditional *F* test results of the ANOVA (not presented herein) for the case of datasets with heterogeneous variances.

**Table 2:** Robust tests of equality of means of the amount of waste collected and distance travelled per shift and discharge per circuit

Test		Statistic^a^	df1	df2	Sig.
Weight per shift	Welch	453.936	18	3648.995	0.000
	Brown-Forsythe	375.235	18	8880.348	0.000
Weight per discharge	Welch	265.110	18	3643.594	0.000
	Brown-Forsythe	167.966	18	5339.223	0.000
Distance per shift	Welch	389.445	18	3651.038	0.000
	Brown-Forsythe	349.615	18	7289.402	0.000
Distance per discharge	Welch	420.359	18	3645.068	0.000
	Brown-Forsythe	717.818	18	4667.023	0.000

^a^Asymptotically *F* distributed

Presenting the results of the post hoc tests would be cumbersome, but the Games-Howell test indicates there are groups of statistically distinct circuits. Based on the Games-Howell results, the number of distinct groups is bigger when analysing the amount of waste collected and distance travelled per shift than per discharge. In terms of weight per shift, the circuits ind_140 and ind_141 are not distinct and are also similar to various other circuits, namely, ind_101, ind_131, ind_150, ind_151, ind_170 and ind_190. The amount of waste collected per shift between the remaining of the circuits is mostly statistically distinct, with the exception of this common ground. In terms of distance travelled, the groups with statistically distinct values are more defined and are the following: (i) circuits ind_150, ind_161, ind_181 and ind_200; (ii) circuits ind_170, ind_171 and ind_191; (iii) circuits ind_100, ind_102, ind_140 and ind_151; (iv) circuits ind_120 and ind_141; (v) circuits ind_111 and ind_131; and (vi) circuits ind_101 and ind_121.

In terms of seasonality, the pattern about the monthly and weekday waste generation was assessed by conducting an ANOVA separately for each month and each weekday (results not presented herein). With the exception of circuits ind_160 and ind_161, there is a statistically significant monthly variation of the amount of waste collected. Globally, the months of July and August are amongst the ones with higher amounts of waste generated and October, November and January amongst the months with lower amounts of waste generated. June, September and December are months with high waste generation in some circuits and average to low in others. This reflects the differences in terms of wealth and tourism intensity in the municipality of Cascais. The weekday seasonality is weaker, with circuits ind_102, ind_130, ind_161, ind_191 and ind_200 showing no statistically significant difference between the amount of waste collected in each weekday. On the other circuits, the pattern is highly variable, with Saturday being one of the weekdays in which the amount of waste is consistently high. Wednesday and Thursday are the days more frequently with low waste generation, but this even more variable between circuits.

Analysing the boxplots of the fuel consumption (Fig. 5), there are some similarities with the boxplots of the distance travelled (Fig. 4), particularly for circuits ind_120, ind_121 and ind_130. However, other circuits (e.g. ind_141, ind_151) are distinct, indicating that other factors in addition to the distance travelled influence the fuel consumption.

The monthly seasonality and circuit differences in terms of total fuel consumption, fuel consumption per amount of waste collected and fuel consumption per distance travel are also statistically significant, but at a weekday basis, there are no statistically significant differences, except in circuits ind_120, ind_140 and ind_161 (Table 3—the Levene test indicates that the homogeneity of variance assumption underlying the ANOVA is violated so the robust tests of equality of means are presented). Circuits ind_102, ind_111, ind_140, ind_150, ind_160 and ind_191 form a group with relatively homogeneous total fuel consumption per shift, with the remaining being statistically distinct in most cases. Regarding the monthly seasonality, there is no clear pattern which months present higher or lower fuel consumption values, with a great variability between the circuits. In some, the summer months (June, July and August) present higher fuel consumptions, in others are winter months (November, December and January), but there are circuits in the months of March, April and May which recorded the highest average fuel consumption values per shift.

**Table 3:** Robust tests of equality of means of the fuel consumption per circuit, month and weekday

Test		Statistic^a^	df1	df2	Sig.
Fuel consumption per circuit					
Total consumption	Welch	321.227	18	3645.790	0.000
	Brown-Forsythe	260.349	18	6219.171	0.000
Consumption per weight	Welch	468.082	18	3635.156	0.000
	Brown-Forsythe	585.612	18	4305.326	0.000
Consumption per distance	Welch	200.593	18	3577.696	0.000
	Brown-Forsythe	143.584	18	5286.827	0.000
Fuel consumption per month					
Total consumption	Welch	4.296	11	3587.206	0.000
	Brown-Forsythe	4.454	11	8320.075	0.000
Consumption per weight	Welch	6.662	11	3579.336	0.000
	Brown-Forsythe	6.609	11	7882.686	0.000
Consumption per distance	Welch	7.363	11	3590.390	0.000
	Brown-Forsythe	7.283	11	8317.799	0.000
Fuel consumption per weekday					
Total consumption	Welch	3.173	6	4399.970	0.004
	Brown-Forsythe	3.228	6	9867.091	0.004
Consumption per weight	Welch	1.599	6	4394.384	0.143
	Brown-Forsythe	1.671	6	9596.667	0.124
Consumption per distance	Welch	0.967	6	4400.943	0.446
	Brown-Forsythe	0.967	6	9902.764	0.446

^a^Asymptotically *F* distributed

### Multidimensional analysis

A regression model was developed to predict the fuel consumption per amount of waste collected, since this was considered the most relevant indicator to evaluate the performance of the service. The total fuel consumption is important for an absolute assessment of the environmental impact of the waste collection service, but it can be calculated knowing the amount of waste collected and the fuel consumption per amount of waste collected. The fuel consumption per distance travelled is less relevant for benchmarking purposes. In addition to the circuit, month and weekday, the specific waste collection truck used was also included as a predictor. The regression model has the following structure:

$$\textrm{Fuel}\ \textrm{consumption}={\beta}_0+{\beta}_1\textrm{Circuit}+{\beta}_2\textrm{Truck}+{\beta}_3\textrm{Month}+{\beta}_4\textrm{Weekday}$$

where *β*_0_ are the regression coefficients. The typical ordinary least squares regression was used and the model obtained (Table 4) is statistically significant and has a R^2^ of 0.66.

**Table 4:** Regression model to predict the fuel consumption per amount of waste collected

Predictor	Coefficient	Std. error	*T*	Sig.	95% confidence interval		Importance
					Lower	Upper	
Intercept	5.606	0.075	75.242	0.000	5.460	5.753	
Circuit (code of the circuit)							
ind_111	− 0.813	0.085	− 9.598	0.000	− 0.979	− 0.647	0.653
ind_120	3.369	0.091	36.953	0.000	3.190	3.548	0.653
ind_121	4.636	0.092	50.221	0.000	4.455	4.817	0.653
ind_130	3.132	0.087	36.007	0.000	2.961	3.302	0.653
ind_131	1.088	0.086	12.587	0.000	0.918	1.257	0.653
ind_141	1.031	0.087	11.873	0.000	0.861	1.201	0.653
ind_150	− 0.275	0.087	− 3.158	0.002	− 0.445	− 0.104	0.653
ind_200	− 1.061	0.106	− 10.022	0.000	− 1.269	− 0.854	0.653
ind_160, ind_161, ind_191	0.251	0.066	3.791	0.000	0.121	0.381	0.653
ind_171, ind_181	− 1.231	0.072	− 17.181	0.000	− 1.372	− 1.091	0.653
ind_102, ind_170	− 0.190	0.074	− 2.581	0.010	− 0.334	− 0.046	0.653
ind_101, ind_151	0.040	0.071	0.560	0.575	− 0.100	0.180	0.653
ind_100, ind_140	0.000						0.653
Truck (number of the truck)							
90	− 0.625	0.121	− 5.155	0.000	− 0.863	− 0.387	0.334
126	− 1.355	0.137	− 9.905	0.000	− 1.623	− 1.087	0.334
186	− 3.071	1.559	− 1.969	0.049	− 6.127	− 0.014	0.334
188	3.999	0.079	50.601	0.000	3.844	4.154	0.334
92, 202	− 0.322	0.075	− 4.276	0.000	− 0.469	− 0.174	0.334
86, 138	− 1.619	0.091	− 17.747	0.000	− 1.798	− 1.440	0.334
137, 236	− 0.882	0.088	− 9.983	0.000	− 1.055	− 0.709	0.334
91, 117, 118, 125	− 0.199	0.080	− 2.495	0.013	− 0.355	− 0.043	0.334
85, 133, 189	0.544	0.064	8.450	0.000	0.418	0.670	0.334
134, 135, 203, 205	0.217	0.057	3.790	0.000	0.105	0.330	0.334
136, 187	0.341	0.072	4.704	0.000	0.199	0.483	0.334
124, 190, 204	0.000						0.334
Month							
July	− 0.370	0.059	− 6.281	0.000	− 0.485	− 0.254	0.012
February, November, December	0.342	0.047	7.307	0.000	0.250	0.433	0.012
January, April, October	0.139	0.043	3.205	0.001	0.054	0.224	0.012
May, June	0.016	0.046	0.347	0.729	− 0.075	0.107	0.012
March, August, September	0.000						0.012
Weekday							
Tuesday, Friday, Saturday	− 0.172	0.032	− 5.460	0.000	− 0.234	− 0.110	0.002
Monday, Wednesday, Thursday, Sunday	0.000						0.002

The model reveals that there are statistically significant differences in terms of waste collection circuit, vehicle used, month and weekday. Since all the predictions of the regression are categorical, the model works in relative terms since there is always a reference category, for which a regression coefficient of 0 is attributed. In the present model, the reference category corresponds to the circuits ind_100 and ind_140, using the truck numbers 124, 190 and 204, during the months of March, August and September and on Mondays, Wednesdays, Thursdays and Sundays. In this reference category, the fuel consumption is 5.606 l/t (corresponds to the intercept). Then, the model corrects this reference value depending on what changes. For instance, maintaining all variables identical to the reference category, except for the weekday that would now be a Tuesday, Friday or Saturday, the fuel consumption would be 5.6 − 0.172 = 5.434 l/t.

**Fig. 6:**
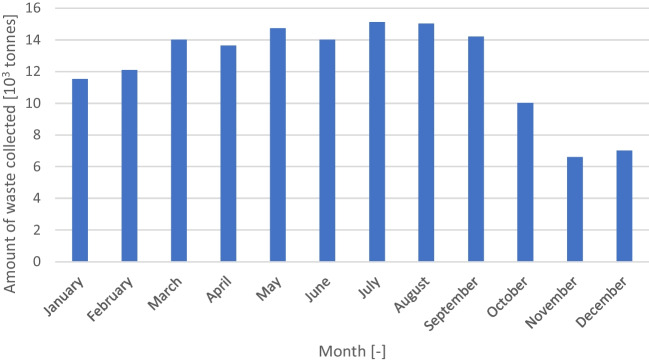
Monthly distribution of the waste generation in 2019

Based on the explanation above, the collection circuits ind_100, ind_140, ind_101 and ind_151 have identical fuel consumption and comprise a line of circuits stretching from north to south roughly at the middle of the municipality. Most of the circuits with higher fuel consumption are located to the west (ind_120, ind_121, ind_130, ind_131 and ind_141), which is the zone less densely urbanised, with a steeper topography (the Sintra mountain is located in this region) and further away from the waste treatment plant (which is located in northeast of the municipality). There is no detectable pattern in terms of performance differences between mixed and segregated waste collection trucks, but it must be regarded that the segregated waste collection trucks are only used in exceptional circumstances. However, considering that the trucks are a proxy of the driver, the driving style has some impact on the fuel consumption. There is a lower specific fuel consumption during the spring and summer months when compared with the autumn and winter months that can be explained by the higher amount of waste generated in these months for the same installed capacity (Fig. 6). This variability of the waste generated is explained by the tourist affluences to Cascais municipality. Regarding the differences between the weekdays, an inverse pattern is observed, with the days with lower waste generation resulting in lower fuel consumption per waste generated. This may be explained by the threshold for the need of more than 1 discharge being surpassed, resulting in a non-linear increase in fuel consumption.

**Fig. 7:**
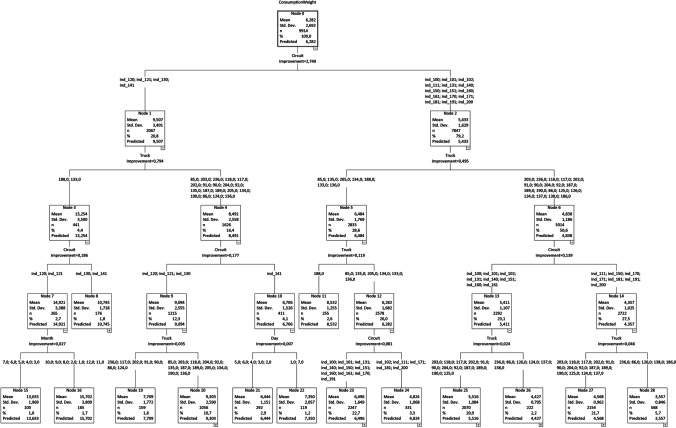
Classification and regression tree (CRT) model

A classification and regression tree (CRT) model was also developed using the same variables and the accuracy is similar (*R*^2^ = 0.67). The tree presented in Fig. 7 does not include all levels since it would make the model impossible to read. Still, it is possible that the circuits are the first differentiating aspect (first level split), followed by the trucks (second level split) and finally the days and months (both in the third level split).

Each node of a CRT model presents the average and standard deviation of the fuel consumption corresponding to the subset of the data indicated by all splits above in the tree (e.g. nodes 1 and 2 separate the collection circuits into two groups, one with an average fuel consumption of 9.5 l/t and the other with an average consumption of 5.4 l/t). The CRT model has two main distinguishing features: (i) not all combinations are possible like in the regression model, with all existing possible combinations in the data being represent by the nodes; and (ii) the results include not only the mean value but also the variability. Regarding the former, it is not possible to simulate the use of the trucks 125, 125, 137, 138 and 189 in circuits ind_120, ind_121, ind_130 and ind_141 because it never occurred in the dataset. Regarding the latter, the node in bottom left corner (node 15) of the tree reports the fuel consumption of the trucks 188 and 133 on the waste collection circuits ind_120, ind_121, ind_130 and ind_141 during the months of March to July, indicating an average of 13.6 l/t and a standard deviation 1.97 l/t.

The results are consistent with the regression model in terms of interpretation, but more comprehensive. The most noticeable difference that can be observed with the CRT model is the fact that the fuel consumption differences between months occur only in circuits ind_102, ind_111, ind_120, ind_121, ind_130, ind_171, ind_181 and ind_200 and the weekdays only affects the fuel consumption on circuit ind_141. This is consistent with the fact that the magnitude of the waste generation pattern variation between the months and the weekdays is not identical in all circuits. The circuit with monthly differences is located mostly in the periphery of the municipality, with exception of circuits ind_102 and ind_111. The explanation for the results of the latter may be related to the fact that they are in one of the most tourist areas, with beaches, casino and other infrastructures that imply a higher variability of the population size and diversity of the waste generation activities. Circuit ind_141 covers the area with most luxury hotels, which explains the weekday variation (occupation rate is higher in some weekdays than other).

Considering as an example the circuit ind_100 and using the collection vehicle 188, the estimated fuel consumption per amount of waste collected estimated using the tree model is 8.5 l/t. Assuming that in a specific shift the amount of waste collected was 12 t (this information is available because the weight discharged at the waste treatment plant is always controlled), the amount of fuel consumed (diesel) is 102 l. Resorting to the results obtained by Sandhu et al. (2015) and Zhou et al. (2016) and performing some reverse engineering assuming proportionality, the emissions can be estimated in 158.8 g of hydrocarbon emissions, 569.7 g of carbon monoxide and 268.8 kg of carbon dioxide.

## Conclusions

Benchmarking and evaluating waste collection utilities based on average performance indicators that do not take into account the specific context in which the operations are carried out may be biased. The present research reveals the substantial variability that may exist in a relatively homogenous medium size municipality. The need to adjust the operation to the waste generation pattern, constrained by shifts lengths, waste treatment plants/transfer stations location, waste collection vehicle characteristics (e.g. capacity, velocity) and urbanisation characteristics (e.g. building typology, spatial distribution, road network), amongst other factors, poses a complex optimization problem that makes it difficult to compare directly with other utilities. The results demonstrate the seasonality of waste generation and the differences between the various circuits that are balanced in terms of average duration to adjust to the worker shift duration (6 h).

The models developed for predicting fuel consumption per amount of waste collected was found to depend on the circuit, vehicle, month and weekday. Considering the variability of the waste generation pattern, the accuracy of the models, *R*^2^ = 0.66 (regression model) and *R*^2^ = 0.67 (CRT model), is similar and quite high. The models provided enable to convert the amount of waste collected into fuel consumed. The regression model is simpler to implement in a spreadsheet, but the tree model allows an easier evaluation of the range of fuel consumption per amount of waste collected in the municipality of Cascais, highlighting critical points to improve fuel efficiency. Additionally, resorting to studies linking fuel consumption with gas emissions, the fuel consumption study carried out provides the missing link from the, usually, available information on the amount of waste collected to the gas emissions. This is valuable to assess the environmental performance of the waste collection service and evaluate the benefits from resorting to alternative fuels or more fuel-efficient vehicles.

## Data Availability

The data is proprietary from EMAC, Cascais Ambiente, and cannot by shared openly. Access must be requested to Mr. André Drumond that will convey to the Administration Board for permission.
